# Challenges in Applying Treat to Target Strategy in Sample of Iraqi Patients with Rheumatoid Arthritis

**DOI:** 10.31138/mjr.32.4.331

**Published:** 2021-12-27

**Authors:** Nizar Abdulateef Jassim, Asal Adnan Redha

**Affiliations:** 1 Department of Medicine, College of Medicine, University of Baghdad, Baghdad, Iraq,; 2 Department of Rheumatology, Baghdad Teaching Hospital, Medical City, Baghdad, Iraq

**Keywords:** Rheumatoid arthritis, treat to target, disease control, disease activity measure, barriers

## Abstract

**Objective:**

To identify barriers and factors that might hamper RA patients from achieving clinical target.

**Patients and methods:**

A total of 100 consecutive RA patients (95 females: 5 males) were included in this retrospective cross-sectional study. Data were collected at one point in time using questionnaire and interview. Demographic data, disease duration, functional classes, medications, and compliance assessment on the current treatments by using the Compliance Questionnaire Rheumatology (CQR19) were recorded for all patients. Achievement of clinical target was defined using the Clinical Disease Activity Index (CDAI; score ≤ 10). The main barriers preventing reaching the clinical target were reported for all the patients with moderate to high disease activity.

**Results:**

Among 100 patients with a recorded CDAI, 58 patients (58%) had not achieved the clinical target (CDAI > 10), for whom the barriers to disease control were recorded. The recorded barriers were drug unavailability/interruption (34.5%), under-treatment (20.7%), insufficient time to assess treatment response to recently initiated DMARDs (12.1%), non- inflammatory musculoskeletal pain (10.3%), had no further drug option available (10.3%), irreversible joint damage (5.2%), and other or non- identified barriers (6.9%). All patients completed CQR-19 items and poor compliance was identified as a predictor of high disease activity (P=0.001). Disease duration had a strong effect on the likelihood of patient response (P=0.035). There was a lower response rate among current smokers (P=0.007). Additionally, functional impairment appeared to be associated with high disease activity (P=0.0001). A significantly larger portion of low disease activity patients were presently on biological treatments (P=0.037) while steroid use had been associated with high disease activity (P=0.03). Age, gender, and Body Mass Index did not predict response to treatment.

**Conclusions:**

This study identified interruption of biologic drugs supply as a large barrier to RA treatment target. The data supported improved outcomes among patients receiving biological treatments. Additionally, certain factors were associated with high disease activity including longer disease duration, functional impairment, smoking, non-adherence, and steroid use.

## INTRODUCTION

Rheumatoid arthritis (RA) is a common systemic autoimmune disease which is characterized by a chronic symmetrical progressive inflammatory polyarthritis.^1^ Rheumatoid arthritis is a worldwide disease affecting adults with an estimated prevalence ranging from 0.5 to 1 %, and a mean annual incidence of 0.02–0.05% in most developed countries.^2^ Treating rheumatoid arthritis (RA) to target (T2T) is quickly becoming a standard of care around the globe after the strategy’s success with diabetes and hypertension.^3^ T2T works by pre-establishing a treatment goal and working to achieve that treatment goal through regular assessment of disease activity and subsequent prescription adjustment when disease persists.^4^ For RA, the T2T goal is typically remission or low disease activity (LDA) for long- standing disease where remission may be unrealistic.^5^ In order to achieve therapeutic goals, this strategy requires adequate patient adherence to physician recommendations. Adherence to T2T recommendations could be influenced by factors relating to the patient, the physician, the disease, medication or other co-morbid conditions.^6^

This study aimed to: a) identify the barriers and challenges to achieve primary target for treatment of rheumatoid arthritis to achieve low disease activity status or state of clinical remission, and b) identify the existence of other factors that might hamper patients from achieving clinical targets.

## METHODS

### Study design

This cross-sectional retrospective study was conducted at the Rheumatology Unit of Baghdad Teaching Hospital in Medical City from July 2018 till March 2019. Informed consent was obtained from each participant included in this study according to the declaration of Helsinki. Ethical approval was obtained from the Ethics Committee in Medical Department, College of Medicine, University of Baghdad.

## PARTICIPANTS

A total of 100 consecutive RA patients (95 females: 5 males) were recruited during their attending the Rheumatology Consultant Clinic for follow-up visits. They were evaluated and enrolled after fulfilling inclusion and exclusion criteria. We included subjects met the 2010 American College of Rheumatology/European League Against Rheumatism classification criteria for RA,^7^ seropositive patients, aged ≥ 18 years, had disease duration ≥ one year and had ≥2 follow up visits (over the last 6 months duration to ensure that every patient had at least two follow-up evaluations). Subjects with history of serious infection that required hospitalization or the use of parenteral antibiotics within the past 12 months, with main comorbidities such as established history of diabetes mellitus, cardiovascular diseases, or past history of neoplastic diseases, pregnant mothers or breastfeeding were excluded from this study. Age, gender, smoking status, body mass index (BMI), disease duration, patient′s global functional status,^8^ and disease activity using Clinical Disease Activity Index (CDAI)^9^ were assessed for each patient. A baseline disease activity during the previous subsequent visits (six months earlier) was obtained from the RA data registry sheets. Previous treatment failure and current treatment (doses and starting date) including corticosteroids, synthetic DMARDs, and therapy with biologic agents were collected. Compliance on the current treatment was assessed by using the Compliance Questionnaire Rheumatology CQR19.^10^Laboratory variables including erythrocyte sedimentation rate (ESR) (measured in mm/hour) at time of evaluation and at baseline, Rheumatoid factor (RF) and Anti-Cyclic Citrullinated Peptide (Anti-CCP) were obtained from routine follow up investigations and RA data registry of Rheumatology Consultant Clinic.

Following data collection by using questionnaire at one point in time, patients were then separated into two groups: moderate or high disease activity (MHDA) comprised of patients with (CDAI > 10) and low disease activity (LDA) (CDAI ≤10). We then examined the data for variables, which may be associated with LDA. All RA patients with a recorded MHDA were re-evaluated for barriers during the last six months (obstacles preventing movement to reach the LDA or remission target goal). These barriers were based on prevalent barriers to disease control in a similar study in Australia.^11^

## STATISTICAL ANALYSIS

The data analysed using Statistical Package for Social Sciences (SPSS) version 25. The data presented as mean, standard deviation and ranges. Categorical data presented by frequencies and percentages. Independent t-test (two-tailed) was used to compare the continuous variables accordingly. Chi square test was used to assess the association between last disease status and certain information. P-value less than 0.05 was considered significant.

## RESULTS

The study group comprised 100 patients. All of them were diagnosed with rheumatoid arthritis. The demographic and the clinical characteristics of patients are shown in **Table 1**.

**Table 1.** Socio-demographic and clinical characteristics of the 100 patients.

**Variable**	**n= 100**	**Percentage (%)**
**Age (years), mean** ± **SD**		**55.2 ± 8.8**
**< 40**	5	5.0
**40 – 59**	54	54.0
**≥ 60**	41	41.0
**Gender**		
**Female**	95	95.0
**Male**	5	5.0
**BMI (kg/m^2^), mean** ± **SD**		**25 kg/m^2^± 2.6**
**Normal**	46	46.0
**Overweight**	49	49.0
**Obese**	5	5.0
**Smoking**		
**Current smokers**	9	9.0
**Non-smokers**	91	91.0
**Disease duration (Years), mean** ± **SD**		**7.8 ± 5.6**
**< 5**	29	29.0
**5 – 10**	49	49.0
**> 10**	22	22.0
**Functional class**		
**FC I**	54	54.0
**FC II**	43	43.0
**FC III**	3	3.0
**Compliance**		
**Good**	66	66.0
**Poor**	34	34.0

(n): number; SD: standard deviation; BMI: Body Mass Index; (kg/m^2^): kilogram per square meter; FC: functional class.

The age of patients was ranging from 30–71 years with a mean of 55.2 years, 95% were women having BMI ranging from 19.5 to 35.6 kg/m^2^ with a mean of 25 kg/m^2^. Most patients were non-smokers (91%), had a disease duration ranging from 1–31 years with a mean of 7.8 years. The highest proportion of patients was found to be in functional class I (54%). Compliance was good in (66%) of patients.

**Figure 1.**
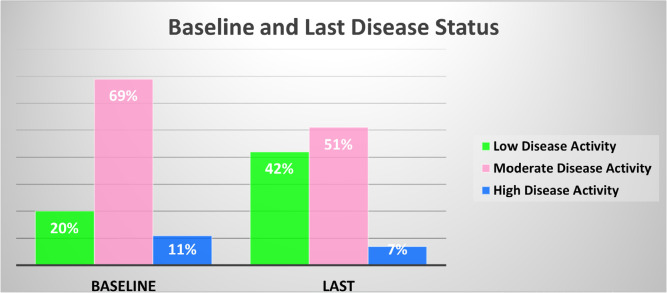
Baseline and last disease status of study patients.

**Figure 1.** shows the baseline and last disease activity status of the patients. We found that 42% of patients met the criteria for low disease activity (LDA) during current evaluation and the prevalence of moderate and high disease status (MHDA) was 58%. We noticed 80% of the included RA patients had a recorded MHDA at baseline (six months earlier from data registry sheets). Long disease duration and current smoking status were significantly associated with MHDA disease activity (P< 0.05). No statistically significant associations (P ≥ 0.05) were observed between last disease activity and other variables (age, gender, BMI) as shown in **Table 2.**

**Table 2.** Association between last disease status and patient demographic characteristics.

**Variable**	**Last Disease Status**		**Total (%)n= 100**	**P – Value**
	**MHDA (%) n= 58**	**LDA (%)n= 42**		
**Age (Years)**				
**< 40**	3 (60.0)	2 (40.0)	5 (5.0)	0.99
**40–59**	31 (42.6)	23 (57.4)	54 (54.0)	
**≥ 60**	24 (41.5)	17 (58.5)	41 (41.0)	
**Mean ± SD**	55.5 ± 9.3	54.8 ± 8.1		0.39
**Gender**				
**Male**	2 (40.0)	3 (60.0)	5 (5.0)	0.35
**Female**	56 (58.9)	39 (41.1)	95 (95.0)	
**BMI (kg/m^2^)**				
**Normal**	25 (54.3)	21 (45.7)	46 (46.0)	0.143
**Overweight**	28 (57.1)	21 (42.9)	49 (49.0)	
**Obese**	5 (100.0)	0 (0)	5 (5.0)	
**Smoking status**				
**Current smokers**	9 (100)	0 (0)	9 (9.0)	**0.007**
**Non-smokers**	49 (53.8)	42 (46.2)	91 (91.0)	
**Disease Duration (years)**				
**< 5**	12 (41.4)	17(58.6)	29 (29.0)	**0.035**
**5–10**	29 (59.2)	20 (40.8)	49 (49.0)	
**>10**	17(77.3)	5 (22.7)	22 (22.0)	
**Mean ± SD**	9.4 ± 6.2	6.2 ± 4.3		**0.027**

n: number; (%): percentage; SD: standard deviation; P-value: probability value (P-value< 0.05 was considered significant); BMI: body mass index; (kg/m^2^): kilogram per square meter; MHDA: moderate and high disease activity; LDA: low disease activity.

There is significant association between poor functional class status, poor compliance with MHDA (P<0.05) as shown in **Table 3.**

**Table 3.** The association between clinical characteristics and disease activity.

**Variable**	**Last Disease Status**		**Total (%) n= 100**	**P – Value**
	**MHDA (%) n= 58**	**LDA (%) n= 42**		
**Functional Class**				
**FC=I**	20 (37.0)	34 (63.0)	54 (54.0)	
**FC=II**	35 (81.4)	8 (18.6)	43 (43.0)	
**FC=III**	3 (100.0)	0 (0.0)	3 (3.0)	**0.0001**
**Compliance**				
**Good**	27 (40.9)	39 (59.1)	66 (66.0)	
**Poor**	31 (91.2)	3 (8.8)	34 (34.0)	**0.001**

n: number; (%): percentage; P-value: probability value (P-value<0.05 was considered significant); MHDA: moderate and high disease activity; LDA: low disease activity; FC: functional class.

Overall, the majority of both LDA and MHDA were currently taking methotrexate and biological treatments (**Table 4**). Notably, patients satisfying the description for LDA were currently using biological agents. There is significant association between steroid use and MHDA (P=0.03). No statistically significant associations (P≥0.05) were observed between last disease activity and other DMARDs (methotrexate, leflunomide, HCQ, and SSZ).

**Table 4.** The association between disease modifying anti-rheumatic drugs and disease activity.

**Variable**	**Last Disease Status**		**P – Value**
	**MHDA (%) n= 58**	**LDA (%) n= 42**	
**Methotrexate**	51 (87.9)	37 (88.1)	0.08
**Leflunomide**	6 (10.3)	2 (4.8)	0.2
**HCQ**	17 (29.3)	5 (11.9)	0.06
**SSZ**	2 (3.4)	5 (11.9)	0.4
**Steroid**	32 (55.2)	10 (23.8)	**0.03**
**Biologic agents**	52 (89.6)	38 (90.5)	0.037

n: number; (%): percentage; P-value: probability value (P-value< 0.05 was considered significant) MHDA: moderate and high disease activity; LDA: low disease activity; HCQ: hydroxychloroquine; SSZ: sulfasalazine.

**Table 5.** How many disease-modifying anti-rheumatic drugs the patients with MHDA had failed.

**n. of patients (%)**	**n.cDMARDs**	**n.bDMARDs**	**n.tsDMARDs**
Twenty-five (43%)	1	0	0
Twenty (34.5%)	2	1	0
Ten (17.2%)	2	2	0
Three (5.2%)	2	3	0
Total= 58 (58%)			

About (43%) of patients with MHDA were previously failed single cDMARDs, while (34.5%) failed 2 cDMARDs and one biological agent, (17.2%) previously failed 2 cDMARDs and 2 biological drugs, however (5.2%) failed 3 biological drugs.

**Figure 2.**
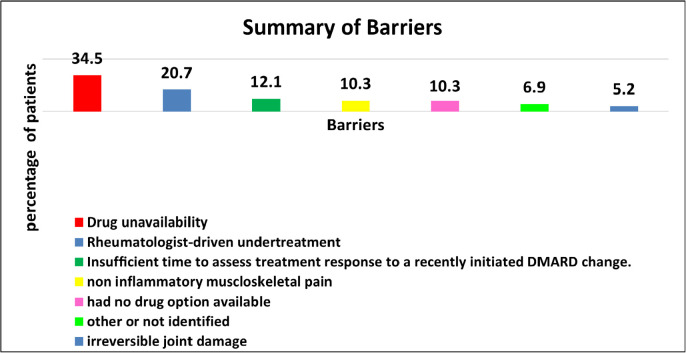
Analysing MHDA barriers to disease control.

The barriers were evaluated as how much they caused disease status to be high or moderate in activity as shown in **Figure 2**. The mostly identified barriers for not-reaching target are drug unavailability/interruptions (34.5%) and under-treatment (20.7%).

## DISCUSSION

Rheumatoid arthritis (RA) continues to be a major health burden that affects quality of life and consumes healthcare resources.^12^ Despite the availability of targeted therapies for RA a wide heterogeneity of treatment strategies and outcome expectations still exists in daily practice, leaving a significant proportion of patients with suboptimal control of their disease.^13^ This retrospective study revealed that a proportion of RA patients were reported as not meeting treatment targets; up to 58% had MHDA while 42% met the criteria for LDA. The major barriers identified in this study could fall into 2 categories: modifiable and fixed barriers. The majority of this group of RA patients with MHDA had modifiable barriers. These included the lack of drug availability (due to interruption of the biological drugs supply) which represented the mostly identified barrier (34.5%). Under treatment to target when continuing existing treatment rather than intensifying therapy was identified in (20.7%). Insufficient time to assess recently initiated DMARD treatment response was considered as a barrier in (12.1%) when more time is required to achieve the effect of a DMARD change (made 1 month ago). In addition, we recognize that (10.3%) of patient had failed anti-TNF and rituximab treatments and the options for additional DMARDs therapy were limited for whom a barrier of having no further drug option available was considered. Fixed barriers were identified in (15.5 %) of the patients. These included non inflammatory musculoskeletal pain from (osteoarthritis, fibromyalgia) and irreversible joint damage were identified in (10.3%, 5.2% respectively); in whom assessing disease activity is difficult as joint swelling and tenderness may be consequences of structural and, hence, irreversible damage. No clear barrier was noted in (6.9%) of the patients where we could allocate the reasons for not reaching targets to other or to none identified.

The same barriers were also reported in the study by Tymms et al.^11^ as they observed that 19.7% of their RA patient-recorded barriers were irreversible joint damage, while drug unavailability represented only 2.1% of the barriers. These differences in barrier frequencies list could be attributed to financial and socioeconomic factors as the most common barrier influencing synthetic DMARDs or biologic prescription. It has been found that poor compliance is associated with poor clinical response with a significant P value (0.001), which could be considered as a further barrier for achieving remission. By this study we assessed the relationship between seven patient characteristics (disease duration, smoking, functional status, medications, gender, age, and BMI) and the response to treatment. Rheumatoid arthritis patients with MHDA had higher mean value of disease duration compared to patients with LDA (P=0.027), and when the patients stratified according to their categories of disease duration, the results demonstrated that the highest percentage (77.3%) of MHDA was seen in patients with disease duration for more than 10 years. Nearly similar results were reported by Anderson et al.^14^ which showed that RA patients with longer disease duration do not respond as well to treatment compared with patients with early disease and that the response rate with any active treatment was higher (53%) for patient with ≤1 year of disease, and lowest (35%) for >10 years (P=0.001). The results of this study showed that smoking was associated with high disease activity with significant P value (0.007). The results demonstrated significant association between disease activity and functional impairment (P value 0.0001), this finding was in agreement by Forslind et al. study^15^ which revealed that HAQ was an independent predictor of remission. When comparing treatment plans of LDA versus MHDA, there was a significantly higher percentage of LDA in patients who were currently on biological medications while steroid use associated with MHDA (P=0.037, 0.03 respectively). Data of current study indicated that disease activity was not associated with gender. This finding was reported by Narváez et al.^16^ which found that sex was not an independent predictor for remission when adjusted for other variables. Our analysis showed no significant association between age and disease activity. Similar result was demonstrated by Jassim et al.,^17^ who showed that age had no significant effect on response to treatment (P>0.05). The results obtained by the current study yielded no significant association between disease activity and BMI. This finding is in accordance with both studies by Pers et al.^18^ and Mirpourian et al.^19^ which revealed no association between BMI and change in DAS28. Although this study represents a unique and novel opportunity to examine controlled RA barriers within the Iraqi population, several limitations are recognized. Our assessment of barriers for not reaching targets was subjective and sometimes the barriers were overlapping/inter-related and as a result there is a potential for misclassification bias, including categorization as modifiable or non- modifiable.

## CONCLUSIONS

Interruption of biologics had been identified as one large barrier to disease control, and non-adherence hampers achieving remission. The data supported an improved outcome among patients receiving biological treatments. Additionally, certain factors emerged as independent predictors of not achieving remission, including longer disease duration, the most disabled patients, smoking, and steroids use.
